# The neuroscience of early intervention: Moving beyond our appeals to fear

**DOI:** 10.1177/0004867421998765

**Published:** 2021-02-26

**Authors:** Lena Palaniyappan

**Affiliations:** 1 Robarts Research Institute, London, ON, Canada; 2 Department of Psychiatry, Western University, London, ON, Canada; 3 The Prevention and Early Intervention Program for Psychoses, London Health Sciences Centre, London, ON, Canada; 4 Department of Medical Biophysics, Western University, London, ON, Canada

**Keywords:** Psychosis, early intervention, neuroscience

## Abstract

This viewpoint is a continuation of the debate on the early intervention movement in psychiatry. The criticisms of Malhi and colleagues have generated some fundamental questions about the priorities of the early intervention movement and the need for further work. In particular, the summons sent to neuroscience need to be more specific in the near future. We may be doing well with what we have, but more directed efforts are needed to purposefully seek what we do not.

Malhi et al. (2020) raise a sharp criticism of the prevailing model of early intervention (EI) in psychiatry. Their main disapproval is that the EI movement seems to put the cart before the horse. They contend that the early intervention idea was ahead of its time for psychiatry; it gathered momentum much earlier before specific interventions for the early phase of illness would become available. As a result, existing interventions offered at later stages of illness have been ‘transposed’, in their words, to an earlier stage. This, as they argue, cannot be considered as early intervention in the true sense.

Malhi et al.’s (2017, 2021) continuous criticism may come across as ‘dirt throwing’ to many of us practising early intervention especially in psychosis. One of the concerns they raise in their recent commentary is on the use of antipsychotics before the onset of a conventionally defined psychotic episode (Malhi et al., 2020). Contrary to their concern, and as pointed out by Woods et al. (2020), this is not the recommended practice at ‘high-risk’ or early psychosis clinics. These programmes do not advocate for inappropriate use of antipsychotics; in fact, the existence of such clinics indeed promotes more appropriate use of antipsychotics for most patients. In fact, the early intervention approach has contributed to a healthy scepticism regarding the length of antipsychotic treatment (Murray et al., 2016). This partly stems from the clearance of the clinical illusion of a relentless progression in psychosis (Zipursky et al., 2013).

One undeniable issue that emerges from Malhi’s arguments is the lack of purpose-built means for early intervention. They argue that this issue has been glossed over for too long, especially as the field contented with using the less than satisfactory interventions of the bygone era, but now at an earlier date in the illness trajectory. This has had one unforeseen consequence in their eyes: the diminution of efforts to ‘fact-check’ early interventions (Malhi et al., 2020). On this issue, Malhi and colleagues stand on firmer ground.

## The headless horseman

To date, the neuroscientific understanding of the effects of early intervention remains minimal. In fact, the ‘neurobiological case’ for EI is often made repeatedly on the basis that EI will reduce progressive brain changes that may otherwise occur (‘active psychosis …. in effect, “bad for the brain” in that it reflected an underlying pathophysiologic process that was progressive unless alleviated by treatment’, from Lieberman et al., 2019). Despite more than 100 years of pursuit, these deleterious progressive brain changes remain the Headless Horseman when treating early psychosis. No one has seen him for sure; but everyone is scared of him and warn others. To make the matters worse, progressive changes may in fact occur secondary to antipsychotic medications, at least in some patients (Ho et al., 2011).

Thus, so far, invoking neuroscientific basis for early intervention has only been a form of well-intended scare tactics. It is important to establish whether the horseman exists, and if indeed, he is headless as the folklore goes. Structural changes are temporally constrained to the early post-onset phase, spatially constrained to selected regions and include both tissue loss and tissue gain (Palaniyappan, 2017). A substantial amount of these changes could indeed be compensatory, representing a reorganisation response to the illness, as argued elsewhere (Palaniyappan and Sukumar, 2020). Thus, the horseman, even if he exists, may not intend the imagined harm.

## Why do latecomers to the clinic get penalised?

One important argument for EI is that latecomers to the clinic do not fare well upon treatment (Drake et al., 2020). EI movement has been focussed on helping people to seek help early, instead of leaving it too late. Promoting early help-seeking is a pragmatic step for any illness that does not resolve spontaneously, and in the case of psychosis may even reduce mortality (Anderson et al., 2018). But why are latecomers less responsive to the same treatment that works better for the more punctual help-seekers? What is the mechanistic basis of this penalty levied on latecomers? This question has to be at the heart of the neuroscience of early intervention, but to date has not been given its due importance (Palaniyappan and Krishnadas, 2020). The answers for this question will move us closer to discovering approaches that are true to the spirit of secondary prevention. Instead, the focus seems to be on turning what started as a targeted secondary prevention to an all-encompassing primary prevention movement (Mei et al., 2020). To this end, demonstrating brain-based differences among the presumed stages of the broadly defined mental illnesses is being seen as an important next step (Shah et al., 2020).

## The three epochs of EI – improving awareness, facilitating access and informing action

The first epoch of EI movement focussed on improving awareness on intervening early for psychosis. The second focussed on improving access to the youth, irrespective of the diagnostic status. The third, from now, needs to focus on discovering appropriate means to provide the so far promised early intervention. This requires being sceptical of the brain-level benefits of early intervention as it is practised currently, that is, asking what good it is to the brain if the currently available interventions are offered during the lead-time gained from early detection. The answer will likely be irrelevant for those who already receive the remarkable social and psychological benefits of early intervention, but very pressing for two other groups – those who continue to come late and those who continue to suffer despite being punctual. These two groups make up too large a number for us to neglect.
